# A pair of 2D chiral Ag(i) enantiomers with dual chiral elements: syntheses, structures, and photoluminescent and chiroptical properties†

**DOI:** 10.1039/d0ra07237k

**Published:** 2020-10-27

**Authors:** Minghui Cui, Ai-Ling Wang, Yingfan Liu, Hongping Xiao, Fengcai Li, Liming Zhou, Shaoming Fang, Xi-Li Li

**Affiliations:** Henan Provincial Key Laboratory of Surface and Interface Science, Zhengzhou University of Light Industry Zhengzhou 450002 P. R. China lixl@zzuli.edu.cn zlming1212@126.com; College of Chemistry & Material Engineering, Wenzhou University Wenzhou 325035 P. R. China

## Abstract

In this work, two new enantiopure bis-monodentate *N*-donor chiral ligands, namely (−)/(+)-2-(4′-pyridyl)-4,5-pinene-pyridine (L_*R*_/L_*S*_), have been designed and synthesized. Using L_*R*_ and L_*S*_ as bridging ligands to react with AgClO_4_, a pair of novel 2D chiral Ag(i) enantiomers formulated as [Ag_2_(L_*R*_)_2_(ClO_4_)_2_]_*n*_ (*R*-1) and [Ag_2_(L_*S*_)_2_(ClO_4_)_2_]_*n*_ (*S*-1) were isolated and characterized. In *R*-1 and *S*-1, each Ag(i) ion is bonded by two N atoms from two different chiral L_*R*_ or L_*S*_ ligands, leading to the formation of 1D right- or left-handed –L–Ag(i)–L– helical chains. Moreover, two adjacent helical chains are further doubly linked by two monodentate ClO_4_^−^ anions through weak Ag–O contacts to form 2D network structures, in which dual chiral elements, *i.e.*, center chirality and helical chirality coexist. Interestingly, each free ligand L_*R*_/L_*S*_ and *R*-1/*S*-1 enantiomers show very different ECD spectra in the solid state and in solution, which are correlated to the intermolecular interactions and molecular structures in each state, respectively. Notably, as a representative, *R*-1 exhibits intense room temperature photoluminescence both in the solid state and in solution with different emission features and mechanisms, while it also shows more intense emission than that of free ligand L_*R*_. In particular, *R*-1 and *S*-1 represent the first examples of 2D Ag(i) chiral coordination polymers (CCPs) supported by ClO_4_^−^ anions, possessing dual chiral elements.

## Abstract 2

2D chiral Ag(i) enantiomeric pair display intense emissions both in the solid state and in solution with very different emission features and mechanisms.

## Graphical abstract

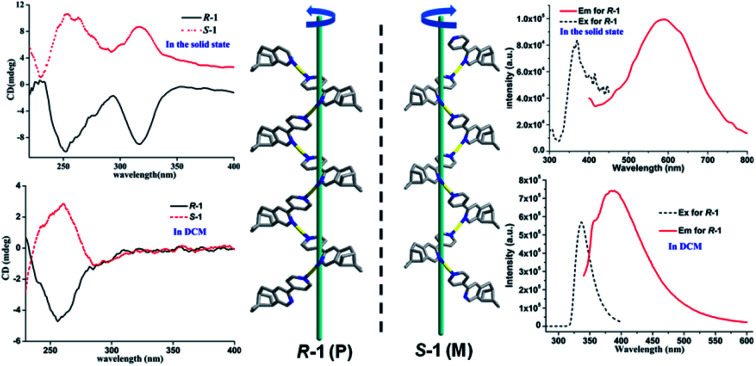

## Introduction

Chirality is a basic feature of life and is ubiquitous in nature, while it also plays an important role in biology, medicine, chemistry and materials science.^1^ Currently, the intense pursuit of chiral coordination polymers (CCPs) is motivated not only by their intriguing structural diversities, but also by their potential applications in asymmetric catalysis, enantioselective recognition and separation, chiroptical probes, nonlinear optical and ferroelectric materials as well as bionic studies.^2^ Typically, three strategies are developed for acquiring CCPs: (i) utilizing homochiral organic bridging ligands or building blocks as linkers to connect adjacent metal centers or metal clusters;^3^ (ii) employing enantiopure agents (solvent, catalyst, co-ligand or template) to induce the formation of chiral frameworks without entering the resulting structures;^4^ (iii) by means of spontaneous resolution upon crystallization without using any chiral sources.^5^ Among them, the second approach remains elusive owing to the fact that how to select a suitable chiral induction agent for given reactants is still unpredictable;^6^ for the third approach, the crystallizing procedures are also uncontrollable. Although spontaneous resolution can result in CCPs for each single crystal, the bulk products generally tend to a racemic mixture without any chiral optical activity.^7^ Thus the first one provides a most direct and reliable method to construct CCPs. In particular, CCPs with more than one chiral elements (central chirality, axial chirality, planar chirality and helical chirality, *etc.*) are of considerable attractive because they not only show fascinating chiral molecular structures, but also may provide multiple active sites for selective catalysis, multifunctional catalysis or concerted catalysis in asymmetric catalytic system.^8^ However, the reported CCPs possessing multiple chiral elements are very scarce, mainly ascribed to the special requirement for the design of chiral bridging ligands.^9^ Ligand plays a crucial role in the formation of single-stranded helix, especially for the aim of constructing CCPs with multiple chiral elements. An effective ligand should not only contain homochiral centers but also possess flexible or semi-rigid structure with at least two sets of independent coordination sites so as to bridge targeted metal ions for the occurrence of helical chirality in CCPs.

**Scheme 1:**
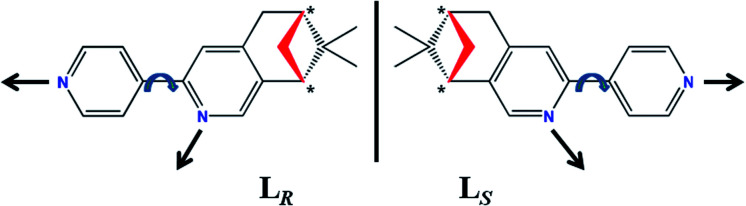
The chemical structures of enantiopure bis-monodentate *N*-donor ligands L_*R*_ and L_*S*_ with possible C–C bond rotations and corresponding orientations of metal–ligand bond vectors.

Over recent years, our group have been devoting to the developments and property investigations of CCPs based on the various homochiral *N*-donor ligands fused with the pinene moiety at 4,5-position of a pyridine ring, involving (−)/(+)-4,5-pinene-2,2′-bipyridine (bidentate chelating ligand, Scheme S1a, ESI†),^2*f*,10^ (−)/(+)-4,5-pinenepyridyl-2-pyrazine (co-existing mono- and bidentate bridging ligand, Scheme S1b, ESI†),^3*c*,11^ and (−)/(+)-2,5-bis(4,5-pinene-2-pyridyl)pyrazine (bis-bidentate bridging ligand, Scheme S2, ESI†).^12^ As an extension of this type of enantiopure *N*-donor ligands with pinene moiety, we have designed and synthesized a new couple of homochiral bis-monodentate *N*-donor enantiomer, (−)/(+)-2-(4′-pyridyl)-4,5-pinene-pyridine (L_*R*_/L_*S*_, Scheme 1), in which each isomer possesses three characteristics: (i) providing two independent monodentate *N*-donor sites for bridging metal centers; (ii) containing two chiral C atoms in the pinene moiety (center chirality, denoted as *); (iii) the inherent structural semi-rigidity allowing for the distinct metal–ligand bond vector orientations by the simple rotation of C–C bond between two different pyridine rings, thus may resulting in the occurrence of single-stranded helix (helical chirality).

On the other hand, Ag(i) ion possesses a wide range of coordination numbers (2–6 or even 7–8) along with various coordination geometries, allowing Ag(i) complexes to exhibit diverse topologies. In addition, Ag(i) ion, with a d^10^ electronic configuration, is a soft Lewis acid and has a high affinity to *N*-donor atom.^13^ The obtained Ag(i)–N complexes usually display interesting emission property and have potential applications as advanced luminescent materials.^14^ For the purpose of constructing new Ag(i) CCPs with multiple chiral elements, we first use enantiopure (−)/(+)-2-(4′-pyridyl)-4,5-pinene-pyridine (L_*R*_/L_*S*_) as chiral bridging ligands to react with AgClO_4_, respectively. Novel chiral Ag(i) enantiomeric pairs formulated as [Ag_2_(L_*R*_)_2_(ClO_4_)_2_]_*n*_ (*R*-1) and [Ag_2_(L_*S*_)_2_(ClO_4_)_2_]_*n*_ (*S*-1) were successfully synthesized and characterized. As expected, *R*-1 and *S*-1 possess dual chiral elements involving chiral C atoms along with the presences of 1D right-handed (P) –L_*R*_–Ag(i)–L_*R*_– helical chains (for *R*-1) and left-handed (M) –L_*S*_–Ag(i)–L_*S*_– helical chains (for *S*-1). The adjacent helical chains are further doubly linked by ClO_4_^−^ anions in monodentate manner through Ag–O contacts, resulting in the formation of 2D Ag(i) CCPs of *R*-1 and *S*-1. In particular, *R*-1 displays intense room temperature photoluminescence both in the solid state and in solution (CH_2_Cl_2_, DCM) with different emission features and emission mechanisms. In addition, electronic circular dichroism (ECD) spectra of each free ligands L_*R*_/L_*S*_ and *R*-1/*S*-1 also present very different patterns in the solid state and in DCM, which are further elucidated in detail.

## Experimental section

### Chemicals and general methods

All raw materials were obtained commercially from J&K Scientific or Tianjin chemical reagent factory and used as received without further purification. All solvents used were of analytical grade. Enantiopure bis-monodentate *N*-donor ligands (L_*R*_ and L_*S*_) were synthesized according to similar procedures.^15^ Elemental analyses for C, H and N were performed on a Perkin-Elmer 2400 Serials II Elemental Analyzer. IR spectra were recorded on a TENSOR27 Bruker spectrophotometer from KBr pellets in the region of 4000–400 cm^−1^. Electronic circular dichroism (ECD) spectra were performed on a Biologic-MOS 500 tester at room temperature. UV-vis absorption spectra were conducted on a UV-4802 Spectrometer. Excitation and emission spectra as well as the decay curve were measured using a FLS980-Combined Fluorescence Lifetime and Steady State Fluorescence Spectrometer (Edinburgh Instrument) with a 450 W xenon lamp and a 100 W μF^2^ microsecond flash lamp as the excitation source. The data were analyzed by software supplied by Edinburgh Instrument. The absolute quantum yield of solid state sample was measured using a demountable integrating sphere from Edinburgh photonics and the values were calculated using its F900 software. Three measurements were made for each sample so that the average value is reported. The estimated error for quantum yields is ±10%.

#### Synthesis of *R*-1

In order to obtain *R*-1, three kinds of solutions were gradually layered in a test tube. The bottom layer was formed by dissolving (−)-2-(4′-pyridyl)-4,5-pinene-pyridine (L_*R*_, 12.5 mg, 0.05 mmol) in DCM (5 mL); the second layer consisted of methanol–DCM (5 mL, 2.5 : 2.5); while the top layer contained AgClO_4_ (10.3 mg, 0.05 mmol) in methanol (5 mL). Then the test tube was covered with parafilm and placed in the dark, and the solvents were allowed to diffuse slowly over three days to afford brown block crystals of *R*-1. Yield: 87% (based on Ag). Elementary analysis (%) calcd for *R*-1 (C_34_H_36_Ag_2_Cl_2_N_4_O_8_, MW = 915.31): C, 44.61; H, 3.96; N, 6.12. Found: C, 44.65; H, 4.08; N, 6.03. IR (KBr, cm^−1^): 2925(m), 1598(m), 1456(m), 1401(m), 1313(m), 1086(s), 951(m), 807(s), 720(s), 619(s).

#### Synthesis of *S*-1

*S*-1 was obtained as brown block crystals by a method similar to that of *R*-1, except that (+)-2-(4′-pyridyl)-4,5-pinene-pyridine (L_*S*_) was used instead of L_*R*_. Yield: 85% (based on Ag). Elementary analysis (%) calcd for *S*-1 (C_34_H_36_Ag_2_Cl_2_N_4_O_8_, MW = 915.31): C, 44.61; H, 3.96; N, 6.12. Found: C, 44.68; H, 3.89; N, 6.06. IR (KBr, cm^−1^): 2923(m), 1597(m), 1457(m), 1403(m), 1312(m), 1085(s), 953(m), 805(s), 721(s), 617(s).

### Single-crystal X-ray crystallography

Single-crystal X-ray diffraction data of *R*-1 and *S*-1 were collected on a Bruker Smart APEX II CCD diffractometer equipped with graphite monochromatized Mo-Kα radiation (*λ* = 0.71073 Å) at 298 K. The data were integrated using the *APEX2* program, with the intensities corrected for Lorentz factor, polarization, air absorption, and absorption due to variation in the path length through the detector faceplate. Multi-scan absorption correction was applied. The structures were solved by direct method using SHELXS program and refined on *F*^2^ by full-matrix least squares using SHELXL-2018. All non-hydrogen atoms were determined from the difference Fourier maps and refined anisotropically. Hydrogen atoms were introduced in calculated positions and refined isotropically using a riding model. The crystallographic data and refinement details for *R*-1 and *S*-1 are listed in Table 1 and selected bond lengths (Å) and angles (°) are presented in Table S1 and S2 (in ESI).†

**Table 1:** Crystallographic data and structure refinement parameters for *R*-1 and *S*-1

	*R*-1	*S*-1
Chemical formula	C_34_H_36_Cl_2_N_4_O_8_Ag_2_	C_34_H_36_Cl_2_N_4_O_8_Ag_2_
Formula weight	915.31	915.31
Crystal system	Monoclinic	Monoclinic
Space group	*P*2_1_	*P*2_1_
*a*/Å	13.2192(7)	13.1734(7)
*b*/Å	10.7130(5)	10.6736(6)
*c*/Å	13.2447(7)	13.2327(7)
*α*/°	90	90
*β*/°	112.909(2)	112.887(2)
*γ*/°	90	90
*V*/Å^3^	1727.73(15)	1714.14(16)
Z	2	2
*D*/g cm^−3^	1.759	1.773
*μ*/mm^−1^	1.346	1.357
GOF	1.038	1.005
*R*_1_ a/w*R*_2_^b^	0.0333/0.0558	0.0373/0.0530
Flack parameter	0.05(2)	0.01(2)

a *R*_1_ = ∑||*F*_o_| − |*F*_c_||/∑|*F*_o_|.

b w*R*_2_ = [∑w(*F*_o_^2^ − *F*_c_^2^)^2^/∑w(*F*_o_^2^)^2^]^1/2^.

## Results and discussion

### Structural characterization

**Fig. 1:**
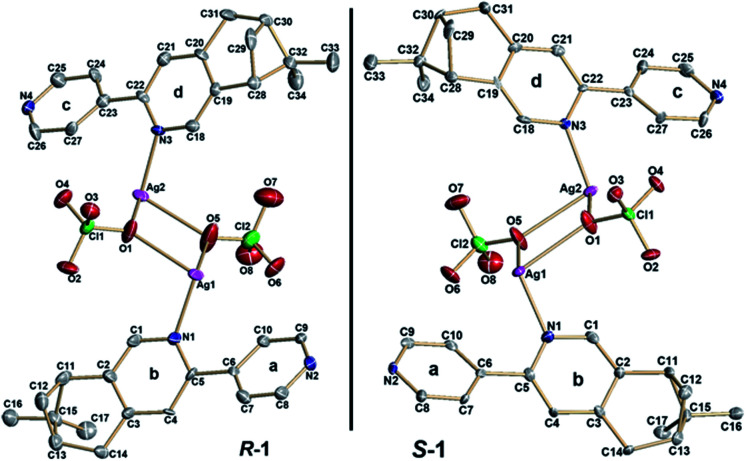
ORTEP representation (50% probability ellipsoids) of the asymmetric units and enantiomeric pairs of *R*-1 and *S*-1 with atom labeling; for clarity, H atoms are omitted.

**Fig. 2:**
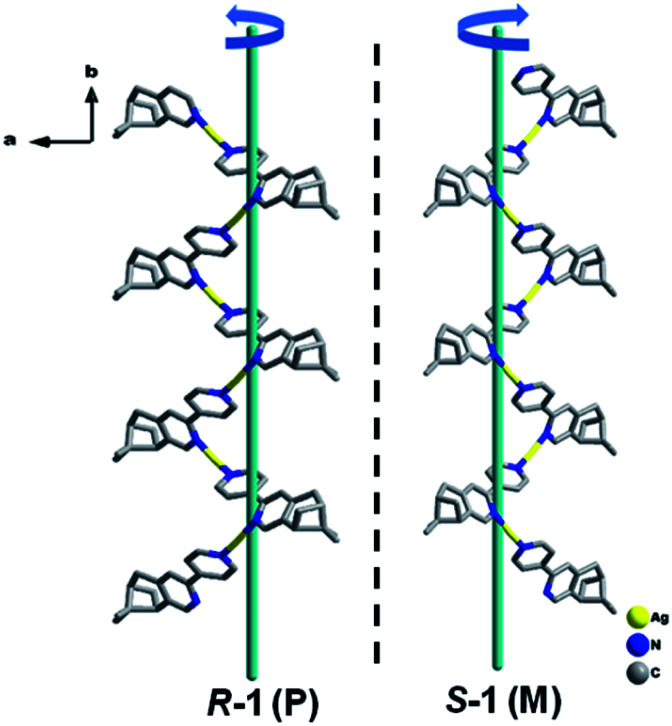
The topologies of 1D right-handed (P) and left-handed (M) 2_1_ helical chains of *R*-1 and *S*-1. H atoms and ClO_4_^−^ anions are omitted for clarity. Color code: Ag, yellow; N, blue; C, gray.

**Fig. 3:**
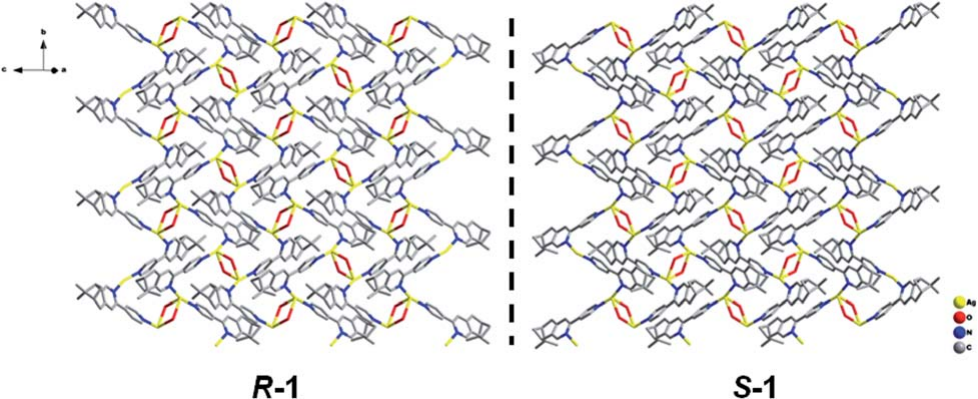
2D chiral sheet network structures of *R*-1 and *S*-1 supported by monodentate [*μ*-O–ClO_3_]^−^ anions. H atoms and uncoordinated O atoms of ClO_4_^−^ anions are omitted for clarity. Color code: Ag, yellow; N, blue; O, red; C, gray.

X-ray crystallographic analysis revealed that both *R*-1 and *S*-1 are enantiomeric pairs crystallizing in noncentrosymmetric space group *P*2_1_ with chiral molecular structures. Since they show similar structure and physical properties, so, only *R*-1 as a representative is described in detail. As shown in Fig. 1, the asymmetric unit of *R*-1 contains two crystallographically independent Ag(i) centers (Ag1 and Ag2), two homochiral bis-monodentate *N*-donor ligands (L_*R*_) and two coordinated ClO_4_^−^ anions. Each Ag(i) center is bounded by two N atoms from two different L_*R*_ ligands with the N–Ag(i)–N angles being 161.0(2) for Ag1 and 161.7(2)° for Ag2, which slightly deviate from linearity due to ClO_4_^−^ anions binding. The dihedral angles between pyridine rings a and b, as well as c and d are found to be 29.5(3)° and 27.5(2)° (Fig. 1), respectively, originating from the rotations of corresponding C5–C6 and C22–C23 bonds. This leads to the formation of 1D –L_*R*_–Ag(i)–L_*R*_– helical chains with a right-handed (P) 2_1_ screw axis in *R*-1, while *S*-1 presents the opposite left-handed (M) helical chains (Fig. 2). Furthermore, two adjacent 1D helical chains are doubly connected by two ClO_4_^−^ anions in monodentate manner through weak Ag–O contacts to create a 2D sheet network structure in the *bc* plane (Fig. 3), which contains two chiral elements, *i.e.*, chiral C atoms and chiral helical chains (P for *R*-1 and M for *S*-1). The interchain Ag⋯Ag distance is 4.30(2) Å, which is longer than the sum of the van der Waals radii of two Ag atoms (3.44 Å), indicating that there are no obvious interchain Ag⋯Ag interactions for *R*-1. In the 2D molecular structure of *R*-1, each Ag-(*μ*-O–ClO_3_)_2_-Ag rhomboid core is bonded to four different L_*R*_ ligands *via* Ag–N bonds. Thus each Ag(i) center is four-coordination with AgN_2_O_2_ polyhedral structure that can be defined as distorted tetrahedral geometry. The distances of Ag–N bonds range from 2.153(7) to 2.199(6) Å, which are obviously shorter than 2.251(5) and 2.215(5) Å found in [Ag_2_(L_2_)(ClO_4_)_2_] with 1D centrosymmetric molecular structure based on monodentate bridging ClO_4_^−^ anions (L = 4,5-diazospirobifluorene).^16^ The average length of Ag–O bonds is 2.635 Å, being much larger than the corresponding value (2.592 Å) obtained in [Ag_2_(L_2_)(ClO_4_)_2_].^16^ Notably, *R*-1 and *S*-1 provide the first examples of 2D Ag(i) CCPs supported by ClO_4_^−^ anions, with dual chiral elements. In addition, the powder X-ray diffraction (PXRD) patterns of the bulk *R*-1 and *S*-1 are in good agreement with the simulated ones based on their single-crystal data, indicating the phase purity of *R*-1 and *S*-1 (Fig. S1 and S2, ESI).†

### Electronic circular dichroism (ECD) spectra of free ligands L_*R*_/L_*S*_ and *R*-1/*S*-1 in the solid state and in solution

**Fig. 4:**
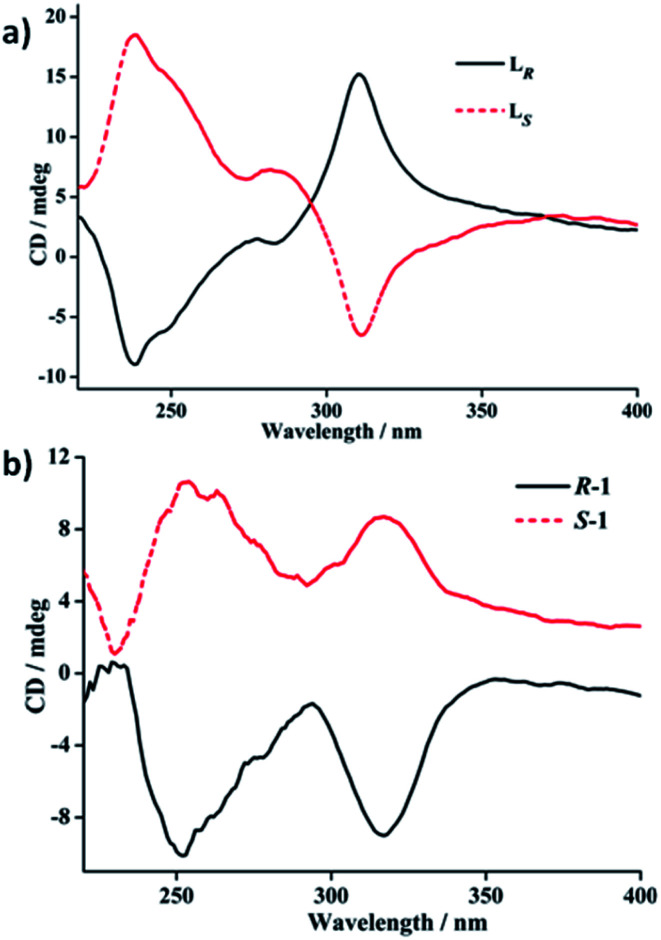
Solid-state ECD spectra of the free ligands L_*R*_/L_*S*_ and *R*-1/*S*-1 enantiomeric pairs based on pressed KCl disks including 1% (wt) of crystal grains at room temperature.

**Fig. 5:**
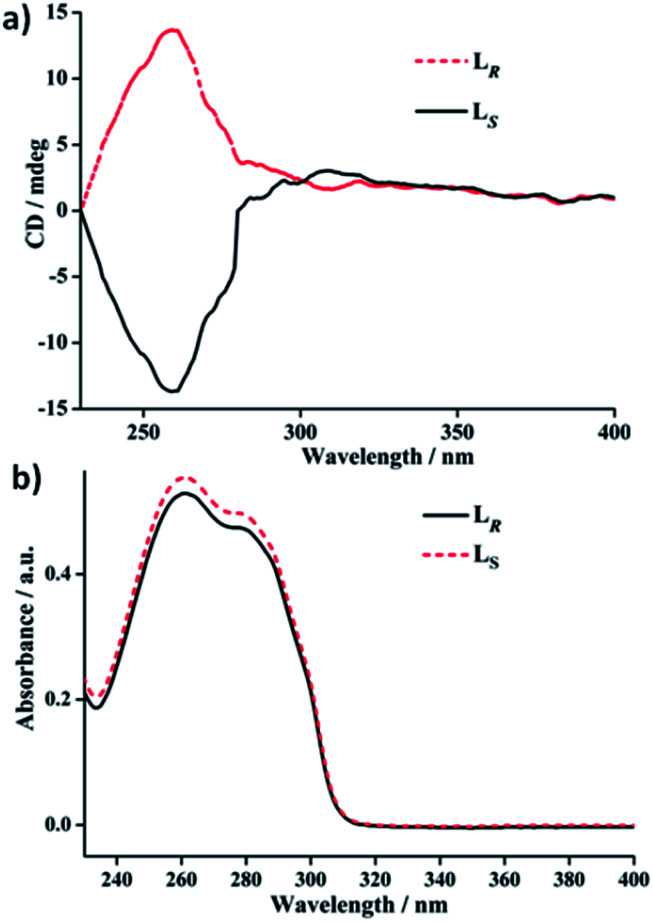
(a) ECD spectra and (b) UV-Vis absorption spectra of free ligands L_*R*_ and L_*S*_ in DCM (1 × 10^−5^ M).

**Fig. 6:**
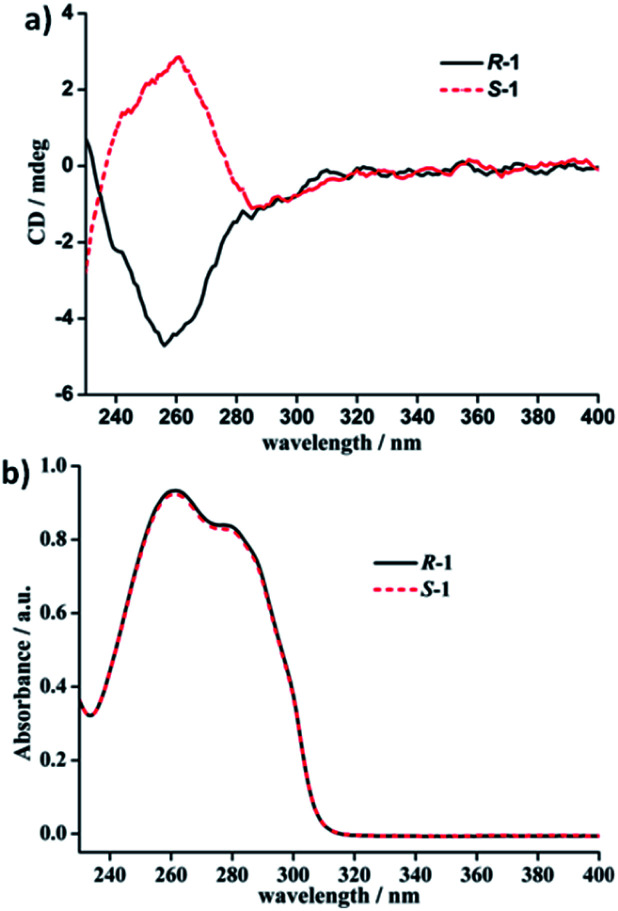
(a) ECD spectra and (b) UV-Vis absorption spectra of *R*-1 and *S*-1 in DCM (1 × 10^−5^ M).

To further verifying the enantiomeric nature and chiral optical property correlated with the structures of each couple of L_*R*_/L_*S*_ and *R*-1/*S*-1 enantiomers, their ECD spectra both in the solid state and in solution (DCM) have been investigated and plotted in Fig. 4–6. In the solid state, the mirror-image ECD signals with the zero-crossing point of the bisignated Cotton effects at 295 nm were observed in free ligands L_*R*_ and L_*S*_ (Fig. 4a). It is noteworthy that the observation of the bisignated curves indicates the presence of exciton coupling between π–π* transition dipole moments in L_*R*_ and L_*S*_.^17^ L_*R*_ displays a positive exciton couplet with two maxima at 310 and 238 nm, while the mirror-image exciton couplet appears with opposite signs at the same wavelengths for L_*S*_. The Davydov splitting of L_*R*_ and L_*S*_ is *ca.* 72 nm, which is much larger than the corresponding value of 28 nm found in a homochiral Dy(iii) tris(β-diketonate) enantiomeric pairs based on enantiopure bidentate *N*,*N*′-donor chelating ligands (Scheme S1a†) similar to L_*R*_ and L_*S*_ in this work.^2*f*^ The mirror-image ECD pattern confirms the chiroptical activity and enantiomeric nature of L_*R*_ and L_*S*_. Interestingly, after the coordination reactions of L_*R*_ and L_*S*_ with AgClO_4_, respectively, the obtained *R*-1 and *S*-1 present very different solid-state ECD pattern without any exciton coupling feature compared to the ECD spectra of free ligands L_*R*_ and L_*S*_ under the same measurement conditions. As shown in Fig. 4b, *R*-1 shows two negative Cotton effects at 252 and 317 nm over the UV spectral range, while the isomer *S*-1 exhibits two mirror-image Cotton effects with opposite signs at the same wavelengths. This also verifies the enantiomeric nature of *R*-1 and *S*-1, which is consistent with the result of crystal structure analysis. In addition, compared to the maxima of Cotton effects for free ligands, *R*-1 and *S*-1 display a bathochromic shift (about 10 nm) at maximum of each Cotton effect, which can be ascribed to the metal-to-ligand charge transfer (MLCT) due to the introduction of Ag(i) ions.^18^

In DCM, the UV-vis absorption spectra of free ligands L_*R*_ and L_*S*_ reveal only a intense absorption band at around 260 nm with a shoulder at *ca.* 280 nm (Fig. 5b), which results from the π–π* and *n*–π* transitions associated with the electrons of the aromatic heterocycle systems. Correspondingly, L_*R*_ and L_*S*_ present the Cotton effects at 260 nm with opposite signs (Fig. 5a). Compared to their solid-state ECD patterns with exciton coupling feature, it is worth mentioning that the solution ECD spectra of L_*R*_ and L_*S*_ only display a set of mirror-image ECD signals at the maxima of their UV-vis absorption bands in DCM. This is mainly due to the absence of intermolecular interaction in L_*R*_ or L_*S*_ caused in aggregation states.^18^ Furthermore, the UV-vis and ECD spectra of *R*-1 and *S*-1 in DCM also were measured and depicted in Fig. 6. The UV-vis absorption spectra of *R*-1 and *S*-1 show the same patterns and the same maximum absorption wavelengths as those for L_*R*_ and L_*S*_ (Fig. 6b), indicating the ligand-centered π–π* and *n*–π* transition absorptions for *R*-1 and *S*-1. Meanwhile, The ECD spectra of *R*-1 and *S*-1 also present the same Cotton effects at 260 nm as those of free ligands L_*R*_ and L_*S*_ in DCM. It is likely that the 2D network structures of *R*-1 and *S*-1 have been destroyed and changed to 1D –L_*R*_–Ag(i)–L_*R*_– and –L_*S*_–Ag(i)–L_*S*_– helical chains, respectively, owing to the dissociation of weak Ag(i)–O bonds in solution.^19^ This is also the reason why the solid-state CD spectra of *R*-1 and *S*-1 are very different from the ones in DCM (see Fig. 6a and 4b).

### Photoluminescent properties of free ligands L_*R*_/L_*S*_ and *R*-1/*S*-1 in the solid state and in solution

**Fig. 7:**
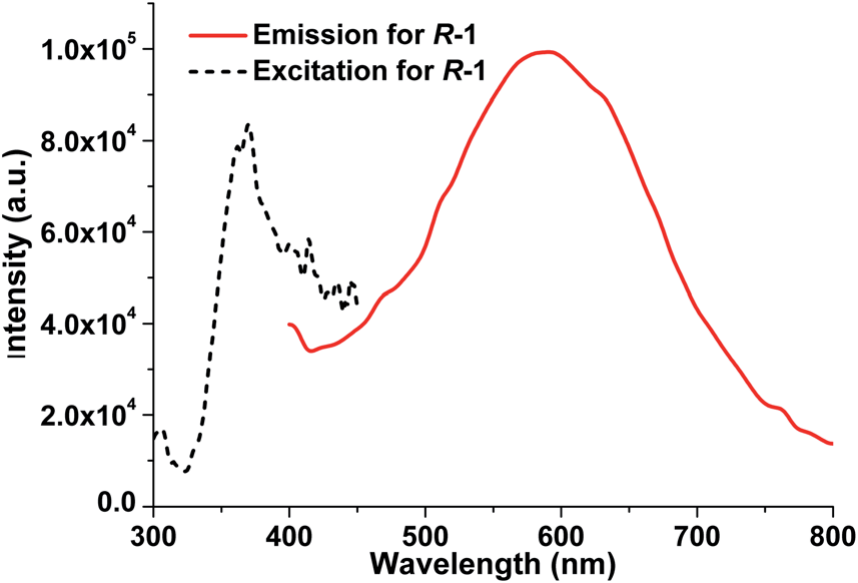
The excitation and emission spectra of *R*-1 in the solid state at room temperature.

It is well recognized that most of the Ag(i)–N complexes usually display weak emission at room temperature owing to the intense spin–orbital coupling of Ag(i) ion,^13*b*,20^ and their luminescent property have been reported almost in the solid state.^13,21^ We investigated the photoluminescent properties of L_*R*_ and *R*-1 both in the solid state and in DCM. In the solid state, for free ligand L_*R*_, no significant luminescence can be detected in the range of 300–750 nm at room temperature. In contrast, upon optimal excitation at 370 nm, *R*-1 exhibits an intense yellow emissive band at 590 nm with a large Stokes shift of *ca.* 220 nm (Fig. 7), which is very rare in documented Ag(i)–N complexes emitting at room temperature.^13*b*^ The emission of *R*-1 in the solid state at 590 nm can be attributed to the electronic transitions from the p orbitals of coordinated N atoms to the 5s orbital of Ag(i) ion, *i.e.*, ligand-to-metal charge transfer (LMCT), together with metal-centered (MC, d–s/d–p) transitions.^13*b*,21*b*^ To further probe the photophysical properties of *R*-1, the luminescent lifetime in the solid state at room temperature was determined from the luminescent decay profile by fitting decay curve to the mono-exponential function (Fig. S3, ESI†), because each Ag(i) center possesses the identical coordination environment. The obtained *τ*_obs_ is 2.60 μs, which is significant longer than 2.5 and 4.89 ns found in [Ag_2_(L_2_)(ClO_4_)_2_]^16^ and [(AgL)ClO_4_] (L = 1,1′-biphenol derivative) with 3D chiral structure,^13*c*^ respectively, as well as other Ag(i) complexes emitting at room temperature.^21*a*,*c*,22^ Moreover, the absolute quantum yield of *R*-1 measured in the solid state reaches 18.70%.

**Fig. 8:**
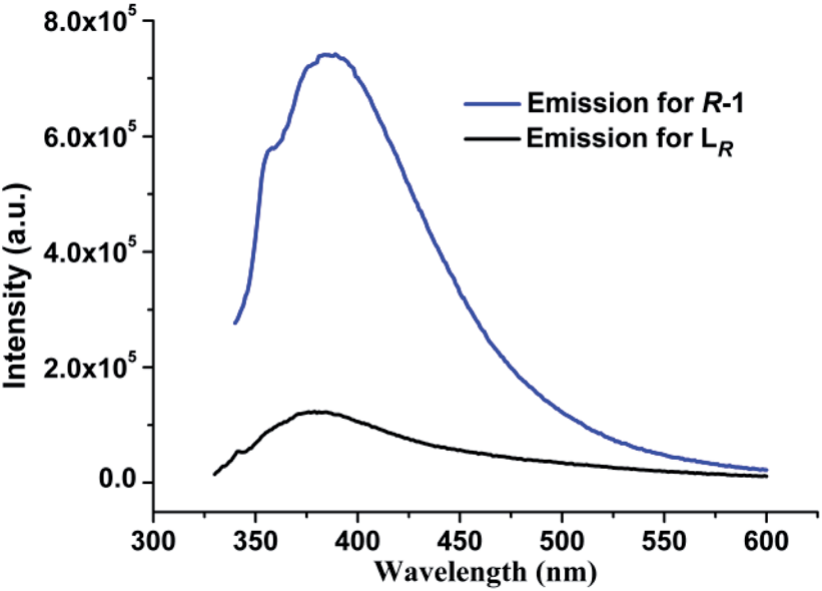
The emission spectra of free ligand L_*R*_ and *R-*1 in DCM (1 × 10^−4^ M) at room temperature.

In DCM, upon excitation at *λ* = 318 nm, the free ligand L_*R*_ displays an emission band centered at 378 nm, arising from the π*–π electronic transitions in aromatic heterocycle system of L_*R*_ (Fig. S4, ESI†). For *R*-1, it presents photoluminescence in DCM with an emission maximum at 387 nm upon excitation at 335 nm (Fig. S5, ESI†), which is slightly red-shifted only 9 nm compared with 378 nm for free ligand L_*R*_. So, the emission mechanism of *R*-1 in DCM can be assigned to the ligand-centered emission.^21*d*^ However, as shown in Fig. 8, the emission intensity of *R*-1 is significant larger than that of free ligand L_*R*_ under the same measurement conditions in DCM. Compared to L_*R*_, the red shift and enhancement of the luminescence from *R*-1 can be mainly attributed to the incorporation of Ag(i) ions, which results in the effective improvement of conformational rigidity of L_*R*_, thus significantly reducing the non-radiative deactivation processes caused by intramolecular vibrations and rotations in L_*R*_.^21*a*^ In addition, *R*-1 displays distinct photoluminescent spectra with different emission mechanism in the solid state and in DCM, originating from the different molecular structures of *R*-1 in these two states as mentioned above.

## Conclusions

A pair of chiral Ag(i) enantiomers have been synthesized based on the use of enantiopure chiral ligands that contain two independent *N*-donor moieties for monodentate bridging. The obtained Ag(i) enantiomers not only possess inherent center chirality from chiral ligands but also present helical chirality created by the incorporation of Ag(i) ions, giving the first examples of 2D Ag(i) CCPs supported by ClO_4_^−^ anions, with dual chiral elements. Due to different intermolecular interactions and molecular structures in the solid state and in solution, enantiopure chiral ligands and 2D chiral Ag(i) enantiomeric pairs display very different ECD spectra in two states, respectively. Notably, the chiral Ag(i) enantiomeric pairs show intense emissions both in the solid state and in solution at room temperature. In addition, our results also demonstrate that the judicious selection of ligand is crucial for constructing CCPs with multiple chiral elements.

## Conflicts of interest

There are no conflicts to declare.

## Supplementary Material

RA-010-D0RA07237K-s001

RA-010-D0RA07237K-s002
