# Supplementary material for: A pair of 2D chiral Ag(i) enantiomers with dual chiral elements: syntheses, structures, and photoluminescent and chiroptical properties
Source: RSC Adv. 2020 Oct 27;10(65):39359–65. doi: 10.1039/d0ra07237k (PMC9057406; doi:10.1039/d0ra07237k)
Supplement: RA-010-D0RA07237K-s001 [file RA-010-D0RA07237K-s001.pdf]

Electronic Supplementary Material (ESI) for RSC Advances.
This journal is © The Royal Society of Chemistry 2020

Supporting information for

A pair of 2D chiral Ag(I) enantiomers with dual chiral elements: syntheses, structures, photoluminescent and chiroptical properties

Minghui Cui,[a] Ai-Ling Wang,[a] Yingfan Liu,[a] Hongping Xiao,[b] Fengcai Li,[a] Liming Zhou,*[a] Shaoming Fang[a] and Xi-Li Li*[a]

[a]Henan Provincial Key Laboratory of Surface and Interface Science,

Zhengzhou University of Light Industry, Zhengzhou, Henan 450002, P. R. China.

E-mail: lixl@zzuli.edu.cn; zlming1212@126.com

[b]College of Chemistry & Material Engineering, Wenzhou University, Wenzhou 325035, P. R. China

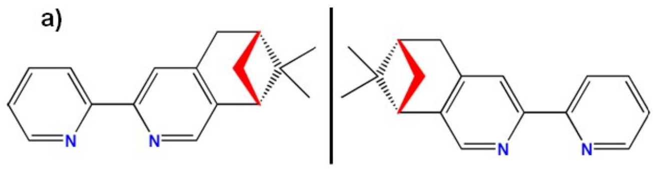

(−)-4,5-pinene-2,2′-bipyridine (+)-4,5-pinene-2,2′-bipyridine

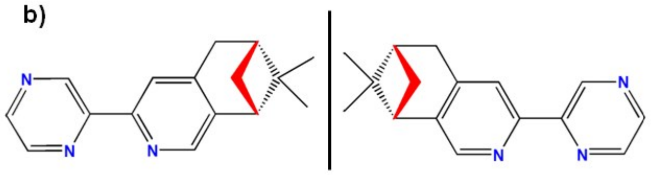

(−)-4,5-pinenepyridyl-2-pyrazine (+)-4,5-pinenepyridyl-2-pyrazine

Scheme S1. The chemical structures of enantiopure *N*-donor chiral ligands with pinene moieties.

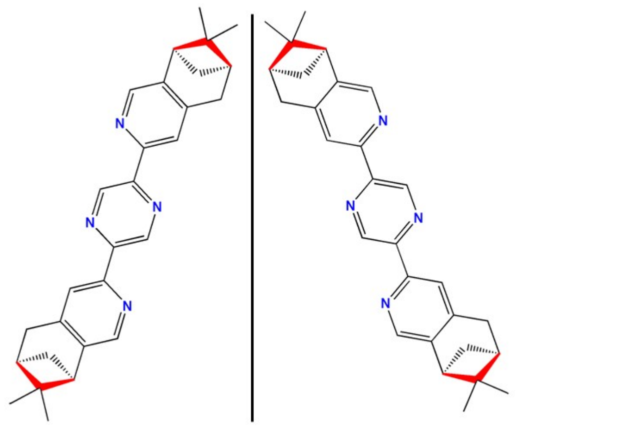

Scheme S2. The chemical structures of enantiopure *N*-donor bis-bidentate chiral ligands with pinene moieties.

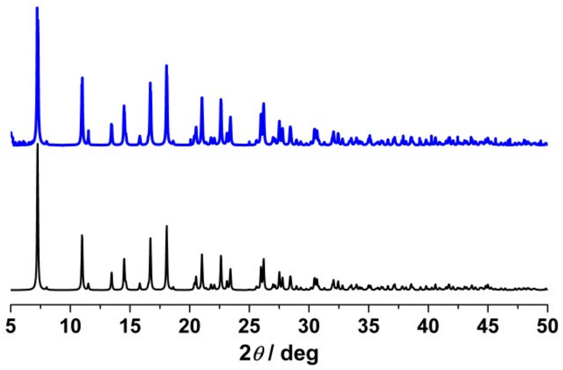

Fig. S1 Powder X-ray diffraction (PXRD) patterns of a simulation based on single crystal analysis of ***R*-1** (black) and as-synthesized ***R*-1** (blue).

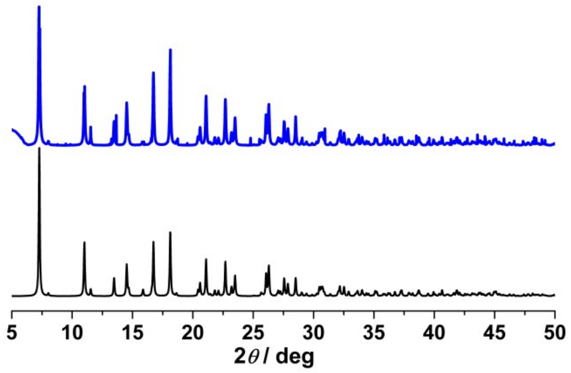

Fig. S2 Powder X-ray diffraction (PXRD) patterns of a simulation based on single crystal analysis of ***S*-1** (black) and as-synthesized ***S*-1** (blue).

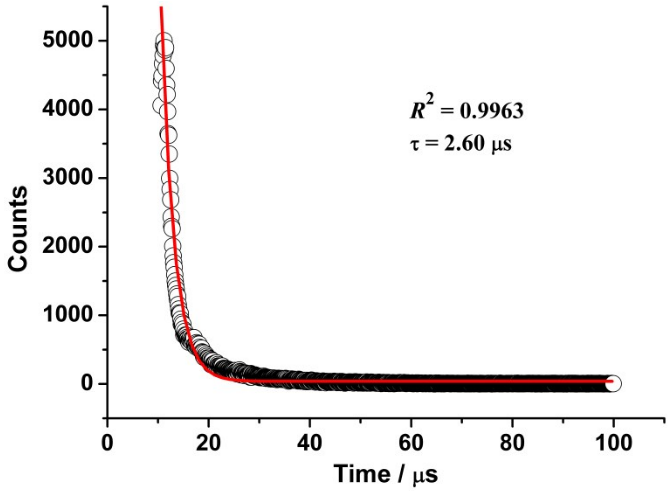

Fig. S3 Decay curves of ***R*-1** with fitted curves (red) in the solid state at room temperature.

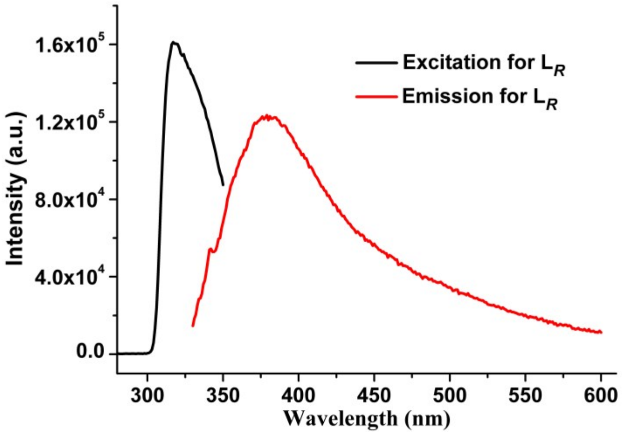

Fig. S4 The excitation and emission spectra of $\mathbf{L}_{\boldsymbol{R}}$ in DCM (1×10^{-4} M) at room temperature.

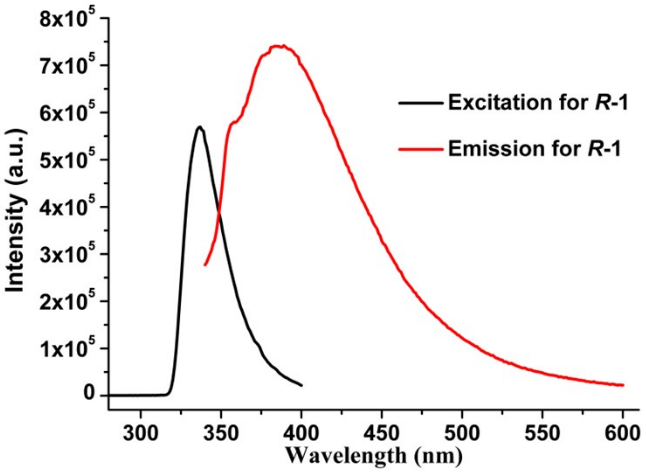

Fig. S5 The excitation and emission spectra of ***R*-1** in DCM (1×10^{-4} M) at room temperature.

Table S1. Selected bond length (Å) and angles (°) for ***R*-1**

Bond lengths			
Ag(1)–O(1)	2.610(6)	Ag(2)–O(1)	2.611(5)
Ag(1)–O(5)	2.693(6)	Ag(2)–O(5)	2.626(7)
Ag(1)–N(1)	2.182(7)	Ag(2)–N(3)	2.199(6)
Ag(1)–N(2)	2.153(7)	Ag(2)–N(4)	2.190(7)
Bond angles			
N(1)–Ag(1)–O(1)	100.7(2)	N(4)–Ag(2)–O(1)	93.1(2)
N(1)–Ag(1)–O(5)	105.4(2)	N(4)–Ag(2)–O(5)	91.5(3)
N(1)–Ag(1)–N(2)	161.0(2)	N(4)–Ag(2)–N(3)	161.7(2)
O(1)–Ag(1)–O(5)	70.17(17)	O(1)–Ag(2)–O(5)	71.2(18)
O(1)–Ag(1)–N(2)	91.5(2)	O(1)–Ag(2)–N(3)	102.8(2)
O(5)–Ag(1)–N(2)	92.4(2)	O(5)–Ag(2)–N(3)	102.2(3)

Table S2. Selected bond length (Å) and angles (°) for ***S*-1**

Bond lengths			
Ag(1)–O(5)	2.678(6)	Ag(2)–O(5)	2.603(8)
Ag(1)–O(1)	2.604(7)	Ag(2)–O(1)	2.592(6)
Ag(1)–N(1)	2.171(8)	Ag(2)–N(3)	2.193(8)
Ag(1)–N(2)	2.161(8)	Ag(2)–N(4)	2.186(8)
Bond angles			
N(1)–Ag(1)–O(5)	105.8(3)	N(4)–Ag(2)–O(5)	92.0(3)
N(1)–Ag(1)–O(1)	100.5(3)	N(4)–Ag(2)–N(3)	161.7(3)
N(1)–Ag(1)–N(2)	161.1(3)	N(4)–Ag(2)–O(1)	93.4(3)
O(5)–Ag(1)–N(2)	91.8(3)	O(5)–Ag(2)–N(3)	101.7(3)
O(1)–Ag(1)–O(5)	69.83(18)	O(1)–Ag(2)–O(5)	71.18(19)
O(1)–Ag(1)–N(2)	92.0(3)	O(1)–Ag(2)–N(3)	102.3(2)
